# Evaluation of the Antibacterial Properties of Iron Oxide, Polyethylene Glycol, and Gentamicin Conjugated Nanoparticles against Some Multidrug-Resistant Bacteria

**DOI:** 10.3390/jfb13030138

**Published:** 2022-09-02

**Authors:** Farah M. Abdulsada, Nehia N. Hussein, Ghassan M. Sulaiman, Amer Al Ali, Muhanad Alhujaily

**Affiliations:** 1 Division of Biotechnology, Department of Applied Sciences, University of Technology, Baghdad 10066, Iraq; 2 Department of Medical Laboratory Sciences, College of Applied Medical Sciences, University of Bisha, 255, Bisha 67714, Saudi Arabia

**Keywords:** pathogenic bacteria, antibacterial activity, biofilm, PEG, Fe_3_O_4_ nanoparticle

## Abstract

Antibacterial resistance is observed as a public health issue around the world. Every day, new resistance mechanisms appear and spread over the world. For that reason, it is imperative to improve the treatment schemes that have been developed to treat infections caused by wound infections, for instance, *Staphylococcus epidermidis* (*S. epidermidis*), *Proteus mirabilis* (*P. mirabilis)*, and *Acinetobacter baumannii* (*A. baumannii*). In this case, we proposed a method that involves mixing the Gentamicin (Gen) with iron oxide nanoparticles (Fe_3_O_4_ NPs) and a polymer (polyethylene glycol (PEG)) with Fe_3_O_4_ NPs. X-ray diffraction (XRD), Fourier-transform infrared spectroscopy (FTIR), energy dispersive X-ray (EDX), scanning electron microscope (SEM), and transmission electron microscope (TEM) were used to characterize Fe_3_O_4_ NPs. Zeta potential and dynamic light scattering (DLS) were also assessed. The antibacterial activity of Fe_3_O_4_ NPs, Fe_3_O_4_ NPs+PEG, Fe_3_O_4_ NPs+Gen, and Fe_3_O_4_ NPs+PEG+Gen composites was investigated. The results showed a significant improvement in the antibacterial activity of nanoparticles against bacterial isolates, especially for the Fe_3_O_4_ NPs+PEG+Gen as the diameter of the inhibition zone reached 26.33 ± 0.57 mm for *A. baumannii*, 25.66 ± 0.57 mm for *P. mirabilis,* and 23.66 ± 0.57 mm for *S. epidermidis*. The Fe_3_O_4_ NPs, Fe_3_O_4_ NPs+PEG, Fe_3_O_4_+Gen, and Fe_3_O_4_+PEG+Gen also showed effectiveness against the biofilm produced by these isolated bacteria. The minimum inhibitory concentration (MIC) of Fe_3_O_4_ NPs for *S. epidermidis* was 25 µg mL^−1^ and for *P. mirabilis* and *A. baumannii* was 50 µg mL^−1^. The findings suggest that the prepared nanoparticles could be potential therapeutic options for treating wound infections caused by *S. epidermidis*, *P. mirabilis,* and *A. baumannii*.

## 1. Introduction

Wound infections are one of the most common causes of wound healing failure [1]. When the skin’s ability to protect itself from bacterial invasion is harmed, a substantial amount of exudate accumulates at the damaged location, which along with body temperature, creates a perfect environment for bacterial growth [2,3,4]. *Staphylococcus epidermidis* is the most common Gram-positive bacteria that can cause a range of ailments when it emerges at wound sites. These bacteria can cause serious infections, ranging from localized skin infections to life-threatening conditions including bacteremia and pneumonia [5]. Gram-negative bacteria (such as *Acinetobacter baumannii* and *Proteus mirabilis*) can colonize wounds and cause persistent infections [6]. The formation of biofilms, which are multicellular communities of microbial species that exhibit specific behavior and greater tolerance to high doses of antimicrobial medications as well as the host’s immune response, is a major problem in infected wounds [7,8]. There has recently been a surge in interest in developing new wound dressings that can speed-up healing and prevent infection [9]. Recent increases in bacterial resistance to antibiotics have resulted in serious health consequences. Bacterial resistance has emerged as one of the most difficult problems confronting the majority of antibiotics around the world. Antibiotic resistance has been the subject of numerous attempts to design safe and efficient treatment methods. Antibiotic resistance in bacteria is caused by a variety of processes, including decreased antibiotic absorption and greater antibiotic efflux from the microbial cell [10].

Scientists have been particularly interested in the subject of nanostructured materials technology in recent years because several forms of metallic or oxide nanomaterials offer appealing features and capabilities. Magnetic nanoparticles’ (MNPs) antibacterial mechanisms are thought to be caused by two factors: The development of reactive oxygen species (ROS) on the NPs’ surfaces causes oxidative stress inside the bacterial cell, resulting in cell death [11]. Iron oxide nanoparticles ranging in size from 1 to 100 nm represent a new trend that is increasingly being developed and of interest for adoption in research related to medical applications. Gram-positive and Gram-negative bacteria cause human disease in general. Different bacterial strains were found to be excellent inhibitors of various synthesized metal oxide nanoparticles because Gram-positive and Gram-negative bacteria have different cell walls, and the activity of metal oxide nanoparticles is directly reliant on the bacterial strain [12].

Iron oxide nanoparticles can interact with bacterial cell membranes via electrostatic contact, causing harmful oxidative stress in the bacterium by the generation of free radicals, known as radical oxygen species (ROS) [13]. To synthesize iron oxide nanoparticles, various approaches have been used, including the chemical method, which is simple, efficient, and tractable and allows the size, composition, and form of the NPs to be controlled. The iron oxides can be synthesized through the co-precipitation method of Fe^2+^ and Fe^3+^ by the addition of a base. The size, shape, and composition of iron NPs synthesized by chemical methods rely upon the type of salt used, Fe^2+^ and Fe^3+^ ratio, pH, and ionic strength [14]. A surfactant or a polymer is frequently added at the time of preparation to prevent the nanoscale particle stabilizer from aggregation. The majority of these polymers have substrate-specific adhesion [15]. MNPs can be synthesized and surface-modified by using polyethylene glycol, poly (vinyl alcohol), poly (lactic-co-glycolic acid), poly (vinyl-pyrrolidone), and poly (ethylene-co-vinyl acetate) [16]. Nanoparticles have a high potential as components of wound dressings as an alternative to antibiotics because they have fewer side effects and are not prone to causing microbial resistance. This makes it possible to use them to inhibit the growth of drug-resistant bacteria [17].

Several review articles are known to deal with various wound healing materials, i.e., nanofibers modified with silver nanoparticles [18], electroconductive films, membranes, hydrogels [19], nanocomposites based on polylactic acid and zinc oxide nanoparticles [20], polysaccharide antibacterial hydrogels [21,22], hydrogels with copper nanoparticles [23], membranes with gold or silver nanoparticles [24], and others. The current study aimed to synthesize Fe_3_O_4_ NPs coated with polyethylene glycol (PEG) and combined with Gentamicin (Gen) antibiotic, and to evaluate their antibacterial, anti-biofilm, determination growth curve, minimum inhibitory concentration (MIC), and minimum bactericidal concentration (MBC) on three types of pathogenic bacteria, including *S. epidermidis*, *P. mirabilis*, and *A. baumannii*.

## 2. Materials and Methods

### 2.1. Materials and Reagents

The chemicals employed in this research were ferrous chloride di-hydrate (FeCl_2_·2H_2_O, MW: 162.78 gmoL^−1^), ferric chloride anhydrous (FeCl_3_, MW: 162.2 gmoL^−1^), ammonium hydroxide (NH_4_OH, MW: 35.04 gmoL^−1^), phosphate buffer solution (PBS), and Gen (MW: 477.59 gmoL^−1^), which were procured from Sigma-Aldrich, St. Louis, MO, USA. Deionized water (D.I) was used. PEG was purchased from Beijing Company, Beijing, China. Mueller-Hinton agar medium, brain–heart infusion broth, and tryptone soy broth (TSB) medium from HiMedia, Thane West, India, were used in this study. The dyes used in this study were crystal violet and Giemsa stain (Sigma-Aldrich, Darmstadt, Germany).

### 2.2. Preparation of Iron Oxide Nanoparticles (Fe_3_O_4_)

The Fe_3_O_4_ NPs were prepared using a co-precipitation process described by Lin and his coworkers [25], with minor modifications. Iron (II) chloride dihydrate (FeCl_2_·2H_2_O) has been used instead of iron (II) chloride tetrahydrate (FeCl_2_·4H_2_O) and iron (lll) chloride anhydrous (FeCl_3_) instead of iron (III) chloride hexahydrate (FeCl_3_·6H_2_O). Briefly, in 50 mL of deionized water, 30 mmol of iron (II) chloride dihydrate (FeCl_2_·2H_2_O) and 45 mmol of iron (III) chloride anhydrous (FeCl_3_) were dissolved. For the synthesis of magnetic iron oxide nanoparticles, two different iron chlorides were used (ferrous and ferric precursor salts). The maintenance of Fe^2+^ and Fe^3+^ molar ratios at exactly 1:2 is very important for the purity of Fe_3_O_4_. Then, under steady stirring, 25 mL of ammonium hydroxide (NH_4_OH) was added. The solution was heated to 80 °C for 1 h with constant stirring. The black precipitated nanoparticles were washed several times with deionized water to remove impurities and collected using an external magnet after the reaction was completed. The iron oxide nanoparticles’ precipitate was dried in a hot-air oven for 24 h at 65 °C.

### 2.3. Coating PEG with Fe_3_O_4_ NPs

The previously prepared iron oxide nanoparticles were mixed with the aqueous solution of PEG in a 1:1 ratio (50 mL of Fe_3_O_4_ NPs aqueous solution with 50 mL of PEG) and placed on a magnetic stirrer for 2 h [26].

### 2.4. Loading of Antibiotic Gen

Gen and Fe_3_O_4_ NPs were made by mixing 100 mL of Fe_3_O_4_ NPs prepared by the above method with a 0.001 M (100 mL) aqueous solution of Gen. To improve the interaction between the antibiotic and the magnetic iron oxide NPs (Fe_3_O_4_), constant stirring under ultra-sonication was used. The sonication was performed using a bath-type digital ultrasonic operating at 20 KHz, 5 W, for 30 min [27].

### 2.5. Preparation of the Gen-PEG-Fe_3_O_4_ NPs

This nanocomposite was made by mixing the Gen aqueous solution (0.001 M) with 20 mL of the prepared PEG-Fe_3_O_4_ NPs and magnetically stirring the solution for 2 h at room temperature to enhance Gen uptake [26].

### 2.6. Characterization of Fe_3_O_4_ NPs

The prepared Fe_3_O_4_NPs were confirmed by using the FT-IR analysis (8000 Series, Shimadzu, Japan) as a good method to investigate the various functional groups in Fe_3_O_4_ NPs. The spectral range of iron oxide nanoparticles was measured between wavelengths of 4000–500 cm^−1^ using X-ray diffraction (XRD-6000, Shimadzu, Japan) to determine crystallinity [28,29]. TEM and SEM-EDX from Zeiss, Jena, Germany, were used to determine the size and morphological features of the NPs, and EDX was used to determine the chemical elements. Their size and stability were determined using zeta potential and DLS (SZ-100-Horiba, Indonesia) [30].

### 2.7. Collection of Bacterial Isolates

Three pathogenic bacterial strains were used in this study. The *S. epidermidis*, *P. mirabilis,* and *A. baumannii* were collected from the microbiology laboratory of Al-Kadhimiya Teaching Hospital and Medical City Hospital in Baghdad, Iraq.

### 2.8. Antibacterial Activity

For studying the effect of Fe_3_O_4_ NPs, Fe_3_O_4_ NP_S_+PEG, Fe_3_O_4_ NPs+Gen, Fe_3_O_4_ NPs+PEG+Gen, and Gen, only on the growth of bacterial isolates, Mueller–Hinton agar medium was prepared. The bacterial suspension in this study was prepared and compared with the standard McFarland tube. The plates were swabbed with selected strains and the dishes were allowed to dry at room temperature. Wells were punched into the agar using a sterilized well cutter. The well was loaded with 80 μL at a concentration of 100 µg mL^−1^ of each of Fe_3_O_4_ NPs, Fe_3_O_4_ NPs+PEG, Fe_3_O_4_ NPs+Gen, Fe_3_O_4_ NPs+PEG+Gen, and Gen. The dishes were incubated at 37 °C for 24 h and the inhibition zone diameter was recorded in millimeters [31,32]. The fold increase area was calculated by the following equation:

(1) Fold increase %=(b−a)/a×100

where (*a*) refers to Fe_3_O_4_ NPs or Gen alone, and (*b*) refers to Fe_3_O_4_ NPs+PEG and Fe_3_O_4_ NPs+Gen.

### 2.9. Effect of Prepared Nanoparticles on Bacterial Biofilm by Tube Method

This approach was carried out by culturing a single colony for 24 h in tryptone soy broth (TSB) medium in test tubes with a volume of 5 mL for each tube and adding 1 mL of each concentration of Fe_3_O_4_ NPs, Fe_3_O_4_ NPs+PEG, Fe_3_O_4_ NPs+Gen, and Fe_3_O_4_ NPs+PEG+Gen (12.5, 25, 50, 100 µg mL^−1^) for each tube. After 24 h of the incubation period, the medium was removed and the tubes were washed with a phosphate buffer solution and dried, then they were dyed with 0.1% of crystal violet dye for 5 min. The dye was removed from the tubes and washed with tap water to remove the rest of the dye, and the tubes were left inverted to dry. The results were recorded as follows: the result was (−) if there was no biofilm production, the result was given as (+) if the biofilm formation was weak, the result (+ +) was given if the biofilm formation was medium, and the result (+ + +) was given if the biofilm formation was dense [33].

### 2.10. Determination of the MIC and MBC

MIC and MBC for Fe_3_O_4_ NPs were calculated. In brief, 0.8 mL of brain–heart infusion broth medium was added to test tubes, then 0.1 mL of Fe_3_O_4_ NPs, Fe_3_O_4_ NPS+PEG, Fe_3_O_4_+Gen, and Fe_3_O_4_+PEG+Gen (12.5, 25, 50, 100 µg mL^−1^) was added. Then, 0.1 mL of suspension for each tested bacteria, *S. epidermidis, P. mirabilis,* and *A. baumannii,* compared to a standard McFarland tube, were added. The tubes were shaken well and incubated at 37 °C for 24 h, and then the results were recorded based on turbidity. Then, 100 μL of the mixture was placed over Mueller–Hinton agar medium and incubated for 24 h at 37 °C, with the finding recorded based on whether there was growth (+) or no growth (−) [34].

### 2.11. Determination of Growth Curve

This assay was performed according to the method of Precious Ayanwale and Reyes-López [35], with some modifications depending on the appearance of a difference in the growth of bacteria treated with different treatments. Soy broth medium was inoculated by adding 0.5 mL of the previously prepared bacterial suspension, then adding 1 mL of Fe_3_O_4_ NPs, Fe_3_O_4_ NPs+PEG, Fe_3_O_4_ NPs+Gen, and Fe_3_O_4_ NPs+PEG+Gen. After an incubation period of 0 to 90 min, 0.2 mL of the suspension was transferred to Mueller–Hinton medium and incubated for 24 h at 37 °C. The measurements were recorded depending on whether there was an appearance of a difference in growth or not.

### 2.12. Statistical Analysis

Statistical measurements of the results were investigated by uploading to SPSS (version 16) software and evaluated using one-way ANOVA at a 0.05 level of statistical significance. The data were presented as mean ± SE. All experiments were carried out in triplicate.

## 3. Results and Discussion

### 3.1. Synthesis of Fe_3_O_4_ NPs

**Figure 1:**
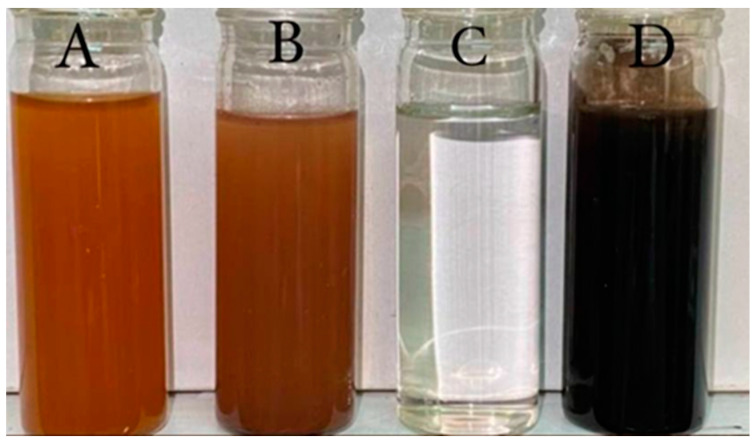
Showing color changing. (**A**) FeCl_2_ and FeCl_3_, (**B**) change color after stirring, (**C**) NH_4_OH added to the mixture then change the color to black as show in (**D**) synthesized Fe_3_O_4_ NPs solution.

**Figure 2:**
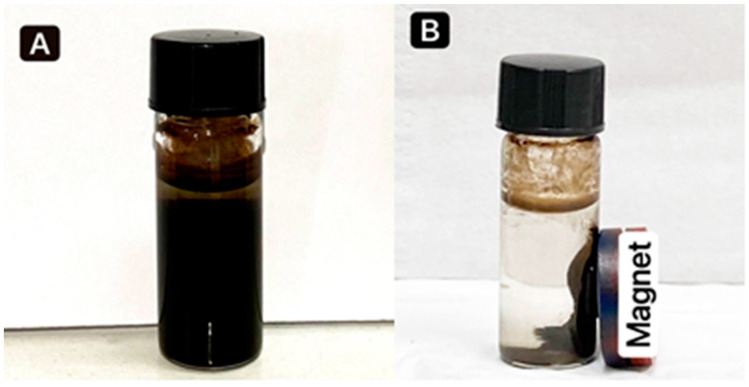
(**A**) Iron oxide nanoparticles (Fe_3_O_4_) without an external magnet, and (**B**) separated Fe_3_O_4_ NPs using an external magnet.

Iron oxide nanoparticles were successfully synthesized by the co-precipitation method, as shown in Figure 1. The composition of Fe_3_O_4_ was confirmed by changing the color to black with ferromagnetic properties, in agreement with [36]. Figure 2A shows that when a magnet is pulled, the NPs are easily distributed by simple shaking. Figure 2B depicts the separation of Fe_3_O_4_ NPs by placing an external magnet near the glass, demonstrating that the Fe_3_O_4_ NPs have magnetic properties. Fe_3_O_4_ NPs coated with PEG (polyethylene glycol) are long polymer chains with many advantages, including non-antigenic, non-immunogenic, and protein-resistant polymers [37]. Furthermore, when PEG was added, it enhanced the compatibility between the nanoparticles and the aqueous solution and reduced toxicity, preventing the surface particles from oxidizing and facilitating storage or transportation. Then, the antibiotic Gen was loaded on synthesized nanoparticles and mixed with the Fe_3_O_4_ NPs+PEG and evaluated for antibacterial activities [38].

### 3.2. Characterization of Prepared Nanoparticles

#### 3.2.1. FTIR Spectrometer

**Figure 3:**
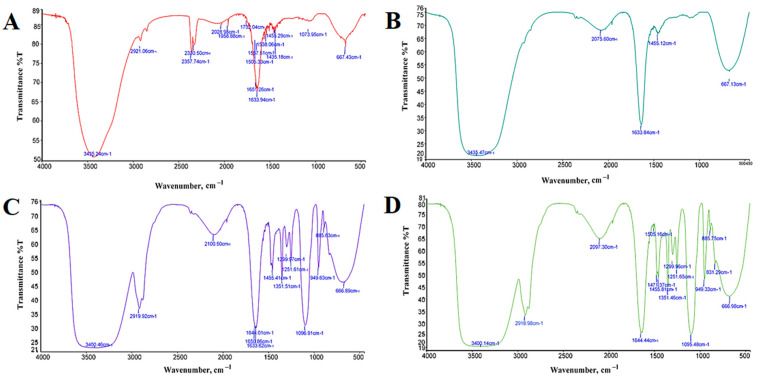
FTIR spectra analysis of (**A**) Fe_3_O_4_ NPs, (**B**) Fe_3_O_4_ NPs+PEG, (**C**) Fe_3_O_4_ NPs+Gen, and (**D**) Fe_3_O_4_ NPs+PEG+Gen.

The FTIR spectra of Fe_3_O_4_ NPs, Fe_3_O_4_ NPs+PEG, Fe_3_O_4_ NPs+Gen, and Fe_3_O_4_ NPs+PEG+Gen are shown in Figure 3 and in Table 1. FTIR analysis was carried out in the wavenumber interval range of 4000–500 cm^−1^. The data plot transmits the wavenumber of infrared light in the form of sharp absorption peaks at certain wavenumbers resulting from the vibration of certain functional groups. In Fe_3_O_4_ NPs, stretching vibration of the Fe-O functional group occurs for absorption of the infrared wavenumber at 667.43 cm^−1^, and the occurrence of a bending vibration of H-O-H at 1633.94 cm^−1^ was identified. The vibration of the O-H (hydroxyl) group is around 3435.24 cm^−1^; in addition, a weak peak recorded at 2921.06 cm^−1^ may be due to the bending vibration of de-ionized water adsorbed on the surface of Fe_3_O_4_ [39]. In Fe_3_O_4_ NPs+PEG, the main absorbance of the ether stretch band is seen at 1096.91 cm^−1^ and the vibration of the O-H group is around 3400.46 cm^−1^. Bending vibrations of -CH_2_ and -CH bands are seen at 1455.41, 1299.97, and 949.63 cm^−1^, respectively. Furthermore, H-O-H bending is seen at around 1650.86 cm^−1^. Fe-O vibration appeared at around 666.89 cm^−1^, while at 2919.92 cm^−1^ due to the OH stretching vibration band [40]. In Fe_3_O_4_ NPs+Gen, the peak at 3435.47 cm^−1^ is attributed to O-H stretching vibrations, and H-O-H bending is seen at around 1633.84 cm^−1^. In addition to the Fe-O vibrating around 667.13 cm^−1^, the C-N stretching of Gen is also visible at 1455.12 cm^−1^ [41]. In Fe_3_O_4_ NPs+PEG+Gen, the main absorbance of the ether stretch band is seen at 1095.48 cm^−1^. Bending vibrations of -CH_2_ and -CH bands are seen at 1471.37, 1299.96, and 949.33 cm^−1^, respectively. Furthermore, Fe-O vibration appears at around 666.98 cm^−1^, H-O-H bending is seen at around 1644.44 cm^−1^, and the vibration of the O-H (hydroxyl) group is at around 3400.14 cm^−1^ [42]. In conclusion, the peaks at around 666 and 667 cm^−1^ are attributed to the stretching vibration of the Fe-O bond, confirming the presence of crystalline Fe_3_O_4_ NPs for all. After coating with the polymer of PEG, new absorption bands at 1455.41 and 1471.37 cm^−1^ for Fe_3_O_4_ NPs+PEG and Fe_3_O_4_ NPs+PEG+Gen are prominent for the stretching vibration of C-H but not for Fe_3_O_4_ NPs. This is ascribable to the absence of carbon in bare Fe_3_O_4_ NPs. Moreover, the covalent grafting of PEG onto nanoparticles was also confirmed by the distinct adsorption peaks around 2919.92 and 2918.98 cm^−1^, which are due to the vibration of methylene of PEG for Fe_3_O_4_ NPs+PEG and Fe_3_O_4_ NPs+PEG+Gen. In Fe_3_O_4_ NPs+Gen, the minor peak at 1455.12 cm^−1^ corresponds to the bending vibration of C-H bonds from methyl groups in Gen [43]. In addition, there is a broadband around 3435.24, 3400.46, 3435.47, and 3400.14 cm^−1^ caused by the stretching vibration of hydroxyl group O-H.

#### 3.2.2. X-ray Diffraction

**Figure 4:**
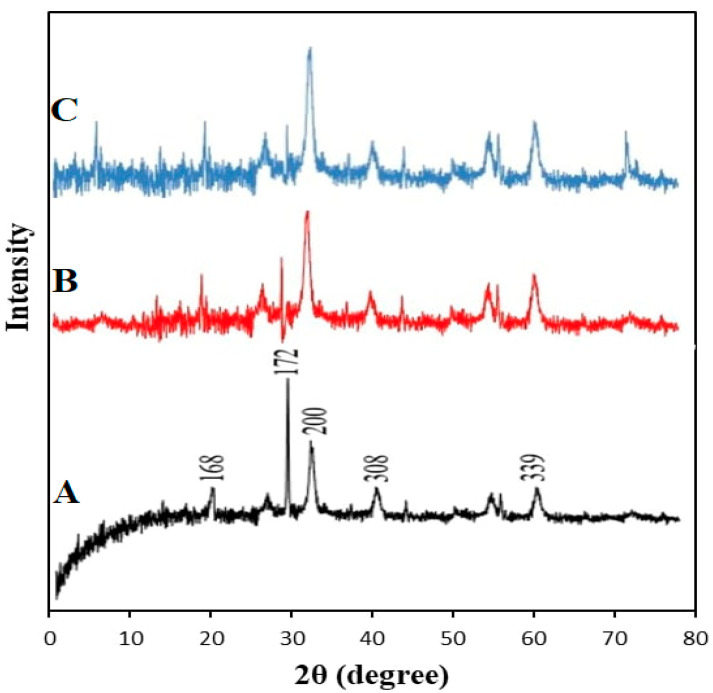
XRD patterns of (**A**) Fe_3_O_4_ NPs, (**B**) Fe_3_O_4_ NPs+PEG, and (**C**) Fe_3_O_4_ NPs+PEG+Gen.

The Fe_3_O_4_ NPs were examined by XRD, which was used to determine the crystal structure and the average size of the particles. As shown in Figure 4, the agreement of the main peaks obtained at (168), (160), (156), (172), (200), (308), (339), and (104) corresponds to the crystalline distance. Bragg reflection was observed at 2θ (θ = diffraction angle) values of 30.4°, 40.44°, 43.31°, 52.91, 57.27°, 58.43°, 62.81°, and 78.02°, respectively. These results confirm that the material examined indicates that the magnetic powders of black color are Fe_3_O_4_ NPs and that they are of high purity. The strong peaks also indicate that Fe_3_O_4_ NPs are pure and have an excellent crystallinity structure. The observed peak amplitude corresponds to the small particle size [44]. The Fe_3_O_4_ NPs+PEG spectra were reduced in intensity because of the addition of PEG, which has amorphous properties. It was discovered that the Fe_3_O_4_ NPs+PEG crystal length was (133), (165), and (254) at 2θ (θ = diffraction angle) values of 35.51°, 57.08°, and 62.77°, respectively. This corresponds with [44]. In the XRD spectra of Fe_3_O_4_ NPs+PEG+Gen, the diffraction peaks were (128), (123), (136), (153), and (249) at 2θ (θ=diffraction angle) values of 30.09°, 35.45°, 43.18°, 56.98°, and 62.61°, respectively [45]. The mean grain size was calculated using the Debye–Scherrer formula, as shown in Equation (2):

(2) D=k λ /β Cos θ 

where D is the mean grain size, k is the Scherrer constant (0.89), λ is the X-ray diffraction wavelength, θ is the Bragg diffraction angle in degrees, and β (in radians) is the full width at half-maximum intensity. The mean grain sizes calculated using this equation for Fe_3_O_4_ NPs, Fe_3_O_4_ NPs+PEG, and Fe_3_O_4_ NPs+PEG+Gen were approximately 37.8, 44.3, and 48.3 nm, respectively.

#### 3.2.3. Scanning Electron Microscopy (SEM)

**Figure 5:**
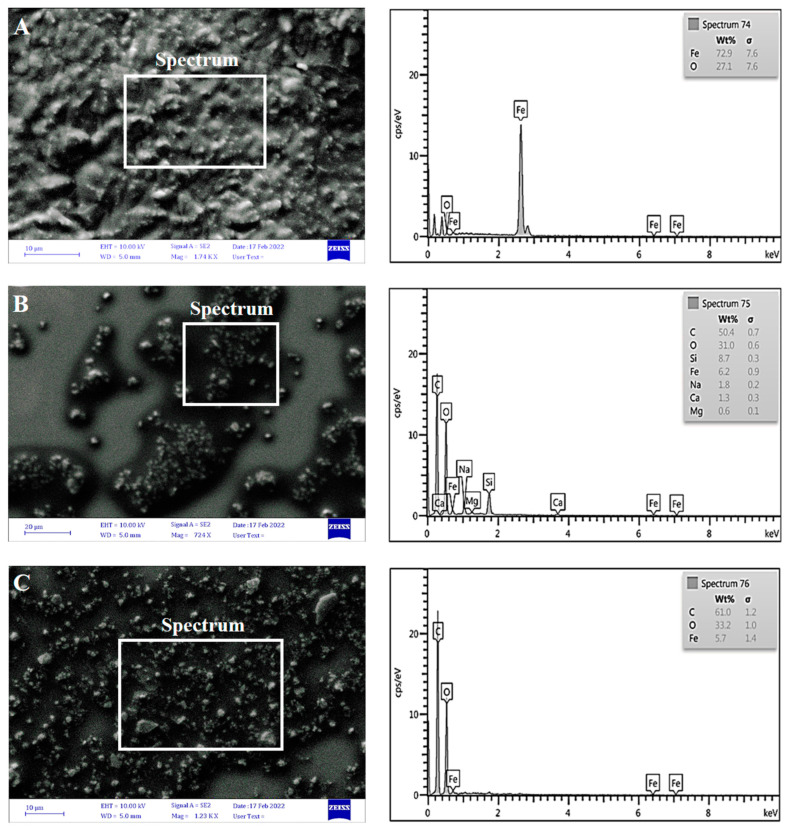
SEM images and EDX of (**A**) Fe_3_O_4_ NPs, (**B**) Fe_3_O_4_ NPs+PEG, and (**C**) Fe_3_O_4_ NPs+PEG+Gen.

The morphology and size of iron oxide nanoparticles were examined by the SEM assay. The image J software was utilized to determine the particle diameter size that is synthesized on the nanometer scale. Micrographs taken at exceptional magnifications are provided in Figure 5, left row. With the observation of particles and aggregation, Fe_3_O_4_ NPs showed a more structural arrangement with a size range of 21.88 to 51.11 nm [46], while Fe_3_O_4_ NPs+PEG showed a size range of 21.09 to 55.54 nm. There were clear differences between Fe_3_O_4_ NPs and after adding PEG, whereby the Fe_3_O_4_ NPs appears to be dispersed, whereas without PEG, the Fe_3_O_4_ NPs appears to be agglomerated [47]. For Fe_3_O_4_ NPs+PEG+Gen, the SEM image size range between 20.51 and 36.23 nm shows that the level of agglomeration decreased because of the Gen coating and the PEG surfactant may absorb selectively onto preferred facets of the crystal. The reduction in particle size after PEG coating was caused by the PEG chains bound to Fe_3_O_4_ NPs, inhibiting crystal growth. The average size of the nanoparticles obtained was less than 100 nm. This is suitable for nano-fluid that must have a size range of 1–100 nm [26].

EDX analysis of the data weights shows that the EDX results of Fe_3_O_4_ NPs were: Fe: 72.9%, has peaks at 0.7, 6.4, and at 7.6 keV, and O: 27.1%, at 0.5 keV. The EDX indicates only Fe (iron) and O (oxygen) elements with no impurities; thus, the EDX evaluation states that the as-synthesized Fe_3_O_4_ NPs are an ideal stoichiometry [48]. On the other hand, the EDX results of Fe_3_O_4_ NPs+PEG were: C: 50.4%, with a peak at 0.7 keV, O: 31.0% at 0.6 keV, Fe: 6.2%, with a peak at 0.9 keV, Si: 8.7%, with a peak at 0.3 keV, Na: 1.8%, with a peak at 0.2 keV, Ca: 1.3%, with a peak at 0.3 keV, and Mg: 0.6%, with a peak at 0.1 keV. The addition of carbon in the Fe_3_O_4_ NPs+PEG can be seen. The main elements in Fe_3_O_4_ NPs are iron and oxygen, whereas when coated with PEG, the percentage of carbon increases [49,50]. The data weights of the EDX results of Fe_3_O_4_ NPs+PEG+Gen were: C: 61.0% at 1.2 keV, O: 33.2% at 1.0 keV, and Fe: 5.7%, with peaks at 1.4, 6.4, and 7.6 keV. The presence of a high-carbon atom in Fe_3_O_4_ NPs+PEG and Fe_3_O_4_ NPs+PEG+Gen was noticed because carbon is an element of PEG and appeared in a high percentage due to the successful coating of PEG with Fe_3_O_4_ NPs. This effect was consistent with that reported by Quevedo et al. [51], as shown in Figure 5, right row.

#### 3.2.4. Zeta Potential Analysis and Average Size Distribution

**Figure 6:**
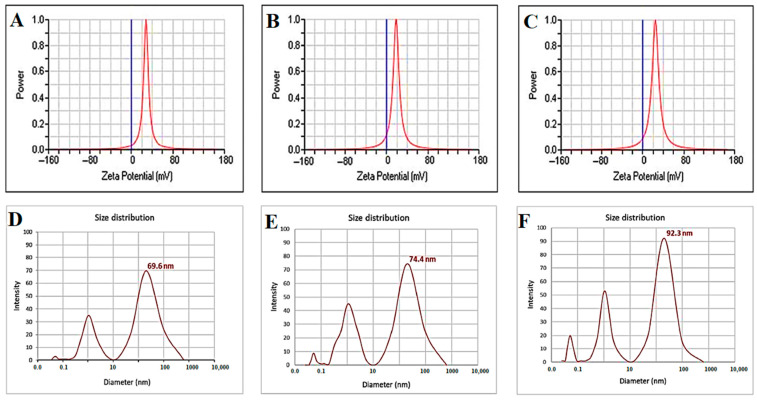
Zeta potential and dynamic light scattering (DLS) of (**A**,**D**) Fe_3_O_4_ NPs, (**B**,**E**) Fe_3_O_4_ NPs+PEG, and (**C**,**F**) Fe_3_O_4_ NPs+PEG+Gen, respectively.

As shown in Figure 6 (top row), zeta analysis was performed to detect the surface charges acquired by iron oxide nanoparticles (Fe_3_O_4_). This test was conducted to get an idea of the stability of the obtained Fe_3_O_4_ nanoparticles. If the particles have a high negative or positive value, the particles will repel each other, and there will be no agglomerating of nanoparticles. On the other hand, if the particles have a small zeta value, no force prevents these particles from aggregation. The value of Fe_3_O_4_ NPs was +28.30 mV due to the OH- ions related to the surface of Fe_3_O_4_ NPs at pH 10, basic medium [52], but the zeta potential of Fe_3_O_4_ NPs+PEG was +18.52 mV. These results revealed that the Fe_3_O_4_ NPs with polyethylene glycol (PEG) could lead to extra pronounced electrostatic stabilization compared to Fe_3_O_4_ NPs [36]. In addition, the zeta potential of Fe_3_O_4_ NPs+PEG+Gen was +24.60 mV, which indicates that the Gen molecule was tightly bound on the Fe_3_O_4_ [53]. As seen in Figure 6 (bottom row), DLS data showed that the average size distribution for Fe_3_O_4_ was 69.6 nm with a PDI (polydispersity) of 0.452, for Fe_3_O_4_ NPs+PEG it was 74.4 nm with a PDI of 0.303, while for Fe_3_O_4_ NPs+PEG+Gen it was 92.3 nm and the PDI was 0.289. The data indicated that the particle sizes increased with the coating polymer and antibiotic and PDI decreased and became monodisperse. This could be due to the generation of a large number of Fe_3_O_4_ NPs coated with PEG, which is an essential factor that can affect particle chemical stability [54].

#### 3.2.5. Transmission Electron Microscopy (TEM)

**Figure 7:**
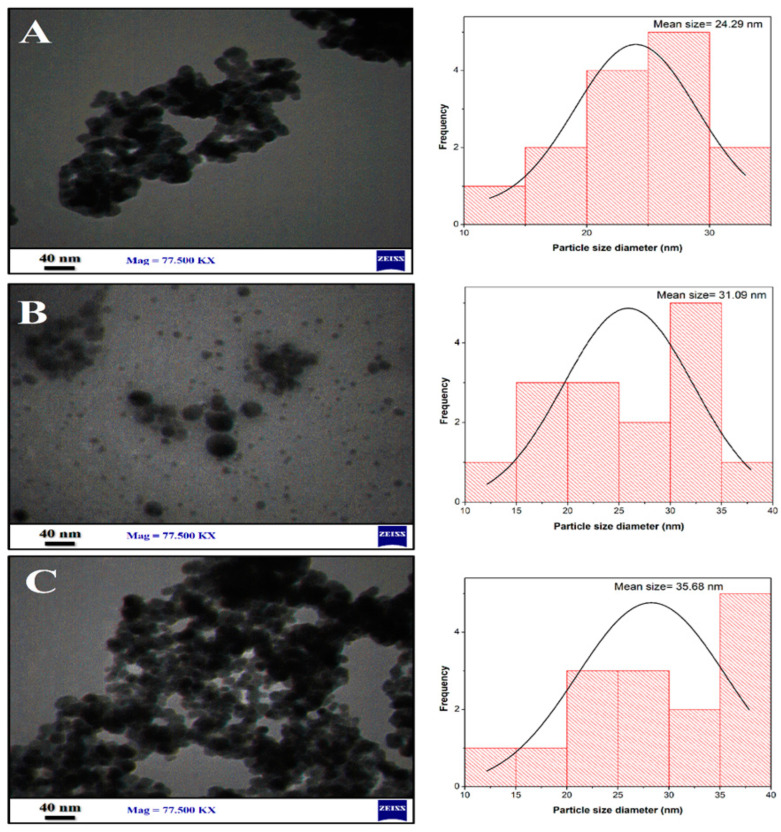
TEM images and histograms of particle size distribution of (**A**) Fe_3_O_4_ NPs, (**B**) Fe_3_O_4_ NPs+PEG, and (**C**) Fe_3_O_4_ NPs+PEG+Gen.

Figure 7 shows the TEM images and particle size distribution of Fe_3_O_4_ NPs, Fe_3_O_4_ NPs+PEG, and Fe_3_O_4_ NPs+PEG+Gen. The image J software was utilized to measure the average mean size of nanoparticles. The obtained Fe_3_O_4_ nanoparticles have a clear and spherical shape with an average size of 24.29 nm, as shown in Figure 7A. Figure 7B shows the addition of PEG, which acts as a stability enhancer and dispersing agent. Moreover, the addition of PEG decreased the agglomeration because PEG modified the surface of Fe_3_O_4_ NPs so that the particle is more monodisperse and uniform, with an average size of 31.09 nm, as seen in Figure 7B. Coating Fe_3_O_4_ nanoparticles with PEG reduced the magnetic interaction between the particles due to their lower magnetism and prevented agglomeration [55]. In addition, Figure 7C shows the TEM images of Gen loaded on the Fe_3_O_4_ NPs+PEG, with an average size of 35.68 nm. The shrinkage in the size when the drug (Gen) was loaded on the nanoparticles could be attributed to the lattice strain generated due to the large Gen molecules, that prevented the nucleation and growth of the Fe_3_O_4_ during the reaction [56].

### 3.3. Antibacterial Activity

**Figure 8:**
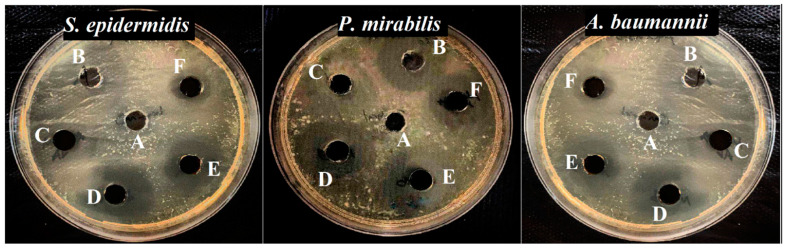
Images of agar plates showing the synergistic effect of Fe_3_O_4_ NPs against the bacterial isolates. (**A**) Control, (**B**) Fe_3_O_4_ NPs, (**C**) Fe_3_O_4_ NPs+PEG, (**D**) Fe_3_O_4_ NPs+PEG+Gen, (**E**) Fe_3_O_4_ NPs+Gen, and (**F**) Gen.

As shown in Table 2 and Figure 8, the antibacterial activity of Fe_3_O_4_ NPs, Fe_3_O_4_ NPs+PEG, Fe_3_O_4_ NPs+Gen, Fe_3_O_4_ NPs+PEG+Gen, and only Gen at a concentration of 100 µg mL^−1^ was tested by the well-diffusion method against Gram-positive bacteria (*S. epidermidis*) and Gram-negative bacteria (*P. mirabilis* and *A baumannii*). The inhibition zone of Gen was 17.5 mm for *P. mirabilis*, 17.6 mm for *A. baumannii*, and 17.3 mm for *S. epidermidis*, whereas the clear inhibition zone for Fe_3_O_4_ NPs+PEG Gen was 25.6 mm for *P. mirabilis*, 26.3 mm for *A. baumannii,* and 23.6 mm for *S. epidermidis* bacteria, further confirming that the Fe_3_O_4_ NPs+PEG+Gen possesses remarkable growth inhibition activity. The results indicate that the Fe_3_O_4_ NPs+PEG+Gen presents noticeable antibacterial activity via a contact-killing mechanism. The interaction between Fe_3_O_4_ NPs+PEG+Gen and bacteria was higher. Iron oxide nanoparticles have both magnetic and paramagnetic properties [57]. The use of an alternating magnetic field allows additional increases in the bactericidal action of Fe_3_O_4_ NPs against *S. epidermidis, P. mirabilis*, and *A. baumannii,* causing cell death and biofilm destruction due to the photocatalytic generation of ROS, and local hyperthermia and vibration damage that occurs under the action of the magnetic field. All the above-mentioned factors lead to the dissociation of bacteria from the biofilm, damage of the bacterial cell wall, membrane rupture, the fusion of different cells with each other, and death [58].

Antibacterial properties were improved when the Gen was coated with Fe_3_O_4_ NPs because it is basically a complex made of closely related aminoglycosides. The main mechanism of action of this drug is the inhibition of protein biosynthesis or genetic translation. When conjugated with iron, Gen forms phospholipids [56]. It forms a layer around the nanoparticles, with the sulphate group forming a covalent bond with the iron [59]. Then the conjugate takes part in the cell annihilation by mainly two mechanisms, namely, interruption of protein synthesis and inducing damage of cell membranes. Thus, it gives rise to a very effective drug carrier [60]. The inhibition zone diameter was 23.6 mm for *P. mirabilis* and *A. baumannii,* whereas it was 21.3 mm for *S. epidermidis*.

Surface modification is also a key way to improve the antibacterial properties of Fe_3_O_4_ NPs. The coated Fe_3_O_4_ NPs with PEG enhanced the antibacterial activity of Fe_3_O_4_ NPs against *P. mirabilis* and *A. baumannii,* with inhibition zones of 21.6 and 22 mm, respectively, whereas 19.6 mm for *S. epidermidis* due to ROS generation [61]. The inhibition zone for Fe_3_O_4_ NPs alone was 18.6 and 20.3 mm for *P. mirabilis* and *A. baumannii*, respectively, and 17.6 mm for *S. epidermidis*. The possible mechanism of the antibacterial activity of the Fe_3_O_4_ NPs can be affected by the occurrence of an electrostatic adsorption potential between the magnetic iron oxide nanoparticles (positive charge) and pathogenic bacteria (negative charge) [62]. This interaction leads to oxidation of the bacterial membrane upon release of the iron ions by the NPs, which are able to interact with the thiol groups of the membrane proteins. Therefore, this process can increase the potential of nanoparticles to induce oxidative stress reactions and produce reactive oxygen species (ROS). The whole process disrupts the function, permeability, and respiration of the cell membrane. Ultimately, it causes cell breakdown and the death of microorganisms [63]. We noticed that the Fe_3_O_4_ NPs+PEG+Gen had the highest inhibition zone on bacterial isolates compared to other types of Fe_3_O_4_ NPs, because when coating with PEG this prevents aggregation between the particles, in addition to Gen that enhanced its antibacterial activity, as described above, so it had the highest inhibition area and was more effective as compared to the other types of Fe_3_O_4_ NPs [61].

### 3.4. Effect of Fe_3_O_4_ NPs on Bacterial Biofilm by Tube Method

The microbes used were developed in the tryptone soy broth medium, and then the medium was removed and the tubes were dyed with crystal violet dye at a concentration of 0.1%. The amount of dye that stained the tubes varied. If the biofilm formation was weak, the result (+) was reported. If the biofilm formation was moderate, the result (+ +) was reported. If the biofilm formation was dense, the result (+ + +) was reported. A biofilm could not be formed in the negative control tube (−). The effect of iron oxide nanoparticles on biofilm formation was studied by observing the binding of crystal violet dye to adherent cells, which directly reflects the effective ability to inhibit biofilm (Table 3). The results showed that a concentration of 100 µg mL^−1^ of Fe_3_O_4_ NPs reduced the biofilm formation of bacterial isolates *S. epidermidis, P. mirabilis*, and *A. baumannii*. In addition, the Fe_3_O_4_ NPs+PEG, Fe_3_O_4_ NPs+Gen, and Fe_3_O_4_ NPs+PEG+ Gen at 100 µg mL^−1^ exhibited a significant reduction in biofilm formation of *S. epidermidis, P. mirabilis,* and *A. baumannii.* The Fe_3_O_4_ NPs+PEG+Gen was more potent in this regard, as shown in Table 3. These findings revealed a substantial difference in biofilm formation after treatment with Fe_3_O_4_ NPs, which inhibited bacterial attachment to the polystyrene surface, resulting in biofilm detachment and lower biofilm absorbance values [64]. The Fe_3_O_4_ NPs+PEG+Gen adhered to the surface of the negatively charged biofilm through electrostatic interactions and disrupted biofilms, causing the death of bacteria within biofilms [61].

The antibacterial and anti-biofilm effects of these nanoparticles, as well as their physical and chemical properties, have been extensively studied. Nanoparticles with small sizes allow to penetrate the biofilm matrix and have a high surface-to-volume ratio, which promotes powerful interactions with microorganisms and allows them to make contact with microbial cells, resulting in biofilm inhibition. Furthermore, the Fe_3_O_4_ nanoparticles inhibited biofilm production via blocking the formation of exopolysaccharide [65].

### 3.5. Determination of Minimum Inhibitory Concentration (MIC) and Minimum Bactericidal Concentration (MBC) for Fe_3_O_4_ NPs

Table 4 and Supplementary Figure S1 show the MIC and MBC against *S. epidermidis*, *P. mirabilis*, and *A. baumannii*. The results showed that the MIC of Fe_3_O_4_ NPs against *S. epidermidis* was 25 µg mL^−1^, while for both *P. mirabilis* and *A. baumannii* it was 50 µg mL^−1^. The MBC of Fe_3_O_4_ NPs was determined depending on the absence or presence of microbial growth on solid media. The results showed that the MBC became higher than the MIC. The MBC for *S. epidermidis* was 50 µg mL^−1^, whereas for *P. mirabilis* and *A. baumannii* it was 100 µg mL^−1^. A similar effect was recorded for Fe_3_O_4_ NPs+PEG against *S. epidermidis*, *P. mirabilis*, and *A. baumannii*. The MIC of Fe_3_O_4_ NPs+Gen for *S. epidermidis* and *A. baumannii* was 50 µg mL^−1^ and for *P. mirabilis* was 25 µg mL^−1^, and the MBC of Fe_3_O_4_ NPs+Gen against *S. epidermidis* and *A. baumannii* was 100 µg mL^−1^ and for *P*. *mirabilis* it was 50 µg mL^−1^. Finally, the MIC of Fe_3_O_4_ NPs+PEG+Gen for *S. epidermidis* and *A. baumannii* was 50 µg mL^−1^, while for *P. mirabilis* it was 25 µg mL^−1^, and the MBC of Fe_3_O_4_ NPs+Gen for *S. epidermidis* and *A. baumannii* was 100 µg mL^−1^ and for *P*. *mirabilis* it was 50 µg mL^−1^.

The nanoparticles (Fe_3_O_4_) generated in this work were found to be more efficient against Gram-negative bacteria such as *P. mirabilis* and *A. baumannii*, but less effective against Gram-positive bacteria such as *S. epidermidis*. In this regard, the findings are more in line with those of Shahzadi and his colleagues [66]. This is due to the thick peptidoglycan layer of Gram-positive bacteria [67]. This is also due to the negative charge of the lipopolysaccharide layer on Gram-negative bacteria’s outer membrane. They can interact more easily with nanoparticles that have a low positive charge since they have a negative charge. Thereby, this interplay might also create a hollow inside the cellular wall and inflict microorganism death. By injuring and breaking membranes and by penetrating into the cytoplasmic membrane, they exhibit antibacterial activity [68].

### 3.6. Determination of Growth Curve

To assess different bacterial growth over time, the results showed that the time required to inhibit the growth of the bacteria *S. epidermidis, P. mirabilis,* and *A. baumannii* by Fe_3_O_4_ NPs, Fe_3_O_4_ NPs+PEG, Fe_3_O_4_ NPs+Gen, and Fe_3_O_4_ NPs+PEG+Gen was 90 min. As shown in Table 5 and Supplementary Figure S2, we noticed that at time zero, there was no effect on bacterial growth, but after 30 min we noticed growth inhibition, and then at a time of 60 min the inhibition increased. At 90 min, little bacterial growth was observed, and this may be attributed to the interaction between these particles and the groups of sulfur and phosphorus found in the bacterial cell membrane because the proteins of the cell membrane are the preferred sites for the work of these particles, which leads to the destruction of the cell and death [69]. NPs were seen to record some inhibition, showing that nanoparticles were able to generate an amount of ROS in soy broth that led to inhibition of bacterial growth. Given that the generation time and lag phase for the bacteria tested are functions of each nanoparticle and the conditions required for bacterial growth and development, there is evidence that time contributes to the inhibition and growth of bacteria when treated with the aforementioned nanoparticles [70].

## 4. Conclusions

We synthesized magnetic iron oxide nanoparticles by the co-precipitation method, coated them with PEG, and then loaded them with Gen antibiotic. These nanocomposites showed a high antibacterial effect on Gram-negative and Gram-positive bacterial strains *S. epidermidis*, *P. mirabilis,* and *A. baumannii*. The antibacterial efficacy of synthesized Fe_3_O_4_ NPs is largely determined by their physicochemical characteristics (shape, size, and chemical composition). Due to their simple diffusion through the bacterial cell wall, Fe_3_O_4_ NPs +PEG+Gen showed excellent antibacterial activity. This is due to the successful role of polyethylene glycol and Gen resulting in enhanced stability of Fe_3_O_4_ NPs through the electrostatic stabilization mechanism of this anionic capping agent. These findings could help in the understanding of the mechanism of iron oxide nanocomposites (free Fe_3_O_4_ NPs, PEG-coated Fe_3_O_4_ NPs, and Fe_3_O_4_ NPs) with PEG and antibiotic Gen against bacterial cell viability and showed that the synthesized nanoparticles could suppress harmful bacterial strains. These results further affirm the promising potential of these nanoparticles and provide a substantial reason for developing this material as an efficient therapeutic option for treating different infections.

## Figures and Tables

**Table 1:** Functional group in Fe_3_O_4_ NPs.

Bond Type	Functional Group	Wavenumber (cm^−1^)			
		Fe_3_O_4_ NPs	Fe_3_O_4_ NPs+PEG	Fe_3_O_4_ NPs+Gen	Fe_3_O_4_ NPs+PEG+Gen
O-H	Hydroxyl	3435.24	3400.46	3435.47	3400.14
C-H	Stretching	-	1455.41	1455.12	1471.37
H-O-H	Bending	1633.94	1650.86	1633.84	1644.44
Fe-O	Ferrous Oxide	667.43	666.89	667.13	666.98

**Table 2:** The antibacterial activity of Gen, and the mixtures of Fe_3_O_4_ NPs, Fe_3_O_4_ NPs+PEG, Fe_3_O_4_ NPs+Gen, and Fe_3_O_4_ NPs+PEG+Gen.

Bacterial Isolates	Inhibition Zone Diameter (mm)				
	Gen	Fe_3_O_4_ NPs	Fe_3_O_4_ NPs+PEG	Fe_3_O_4_ NPs+Gen	Fe_3_O_4_ NPs+PEG+Gen
***S. epidermidis***	17.33 ± 0.57	17.66 ± 0.57	19.66 ± 0.57	21.33 ± 1.15	23.66 ± 0.57
***P. mirabilis***	17.51 ± 0.57	18.66 ± 0.57	21.66 ± 0.57	23.66 ± 0.57	25.66 ± 0.57
***A. baumannii***	17.66 ± 0.57	20.33 ± 0.57	22.00 ± 0.46	23.66 ± 0.57	26.33 ± 0.57

**Table 3:** Effect of prepared nanoparticles on biofilm formation.

Concentrations (µg mL^−1^)																
Bacterial Isolates	Fe_3_O_4_ NPs				Fe_3_O_4_ NPs+PEG				Fe_3_O_4_ NPs+Gen				Fe_3_O_4_ NPs+PEG+Gen			
	100	50	25	12.5	100	50	25	12.5	100	50	25	12.5	100	50	25	12.5
***S. epidermidis***	−	+	+	+	−	+	+ +	+ +	−	−	+	+	−	−	−	−
***P. mirabilis***	−	+	+	+	−	+	+	+ +	−	+	+	+	−	-	+	+
***A. baumannii***	−	+	+ +	+ + +	−	+	+ + +	+ + +	−	+	+	+ +	−	+	+	+

**Note:** (+) = weak biofilm, (+ +) = moderate biofilm, (+ + +) = dense biofilm, and (−) = no biofilm.

**Table 4:** MIC and MBC of bacterial isolates.

Concentrations (µg mL^−1^)								
Bacterial Isolates	A		B		C		D	
	MIC	MBC	MIC	MBC	MIC	MBC	MIC	MBC
***S. epidermidis***	25	50	25	50	50	100	50	100
***P. mirabilis***	50	100	50	100	25	50	25	50
***A. baumannii***	50	100	50	100	50	100	50	100

(**A**) Fe_3_O_4_ NPs, (**B**) Fe_3_O_4_ NPs+PEG, (**C**) Fe_3_O_4_ NPs+Gen, (**D**) Fe_3_O_4_ NPs+PEG+Gen.

**Table 5:** Effect of time on bacterial growth.

Time (min)																
Bacterial Isolates	Fe_3_O_4_ NPs				Fe_3_O_4_ NPs+PEG				Fe_3_O_4_ NPs+Gen				Fe_3_O_4_ NPs+PEG+Gen			
	Zero	30	60	90	Zero	30	60	90	Zero	30	60	90	Zero	30	60	90
***S. epidermidis***	+ + + + +	+ + +	+ +	+	+ + + + +	+ +	+ + +	+	+ + + + +	+ + +	+ +	+	+ + + + +	+ + +	+ +	+
***P. mirabilis***	+ + + + +	+ + +	+ +	+	+ + + + +	+ + +	+ +	+	+ + + + +	+ + +	+ ++	+	+ + + + +	+ + +	+ +	+
***A. baumannii***	+ + + + +	+ + +	+ +	+	+ + + + +	+ + +	+	+	+ + + + +	+ + +	+ ++	+	+ + + + +	+ + +	+ +	+

Note: (+ + + + +) very dense growth, (+ + +) dense growth, (+ +) medium growth, (+) little growth.

## Data Availability

All the data were provided in the manuscript.

## Supplementary Materials

The following supporting information can be downloaded at: https://www.mdpi.com/article/10.3390/jfb13030138/s1, Figure S1: Minimum inhibitory concentration and minimum bactericidal concentrations for a bacterial isolates. (A) Fe_3_O_4_ NPs, (B) Fe_3_O_4_ NPs+PEG, and (C) Fe_3_O_4_ NPs+PEG+Gen; Figure S2: Determination effect of nanoparticles on microbial growth curve of bacterial isolates. (A) Fe_3_O_4_ NPs, (B) Fe_3_O_4_ NPs+PEG, and (C) Fe_3_O_4_ NPs+PEG+Gen.
