# Supplementary figures and images for: Evaluation of the Antibacterial Properties of Iron Oxide, Polyethylene Glycol, and Gentamicin Conjugated Nanoparticles against Some Multidrug-Resistant Bacteria
Source: J Funct Biomater. 2022 Sep 2;13(3):138. doi: 10.3390/jfb13030138 (PMC9503097; doi:10.3390/jfb13030138)

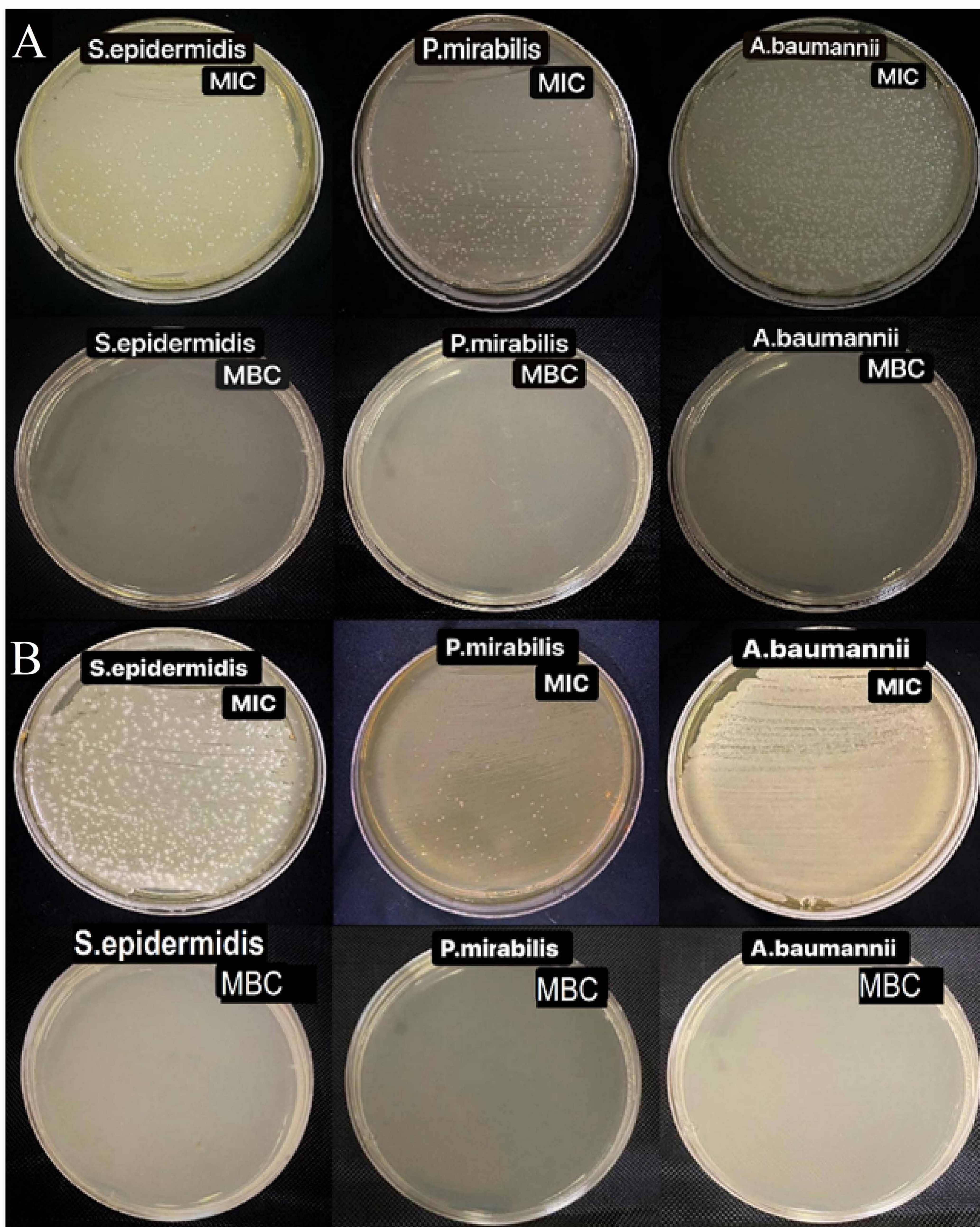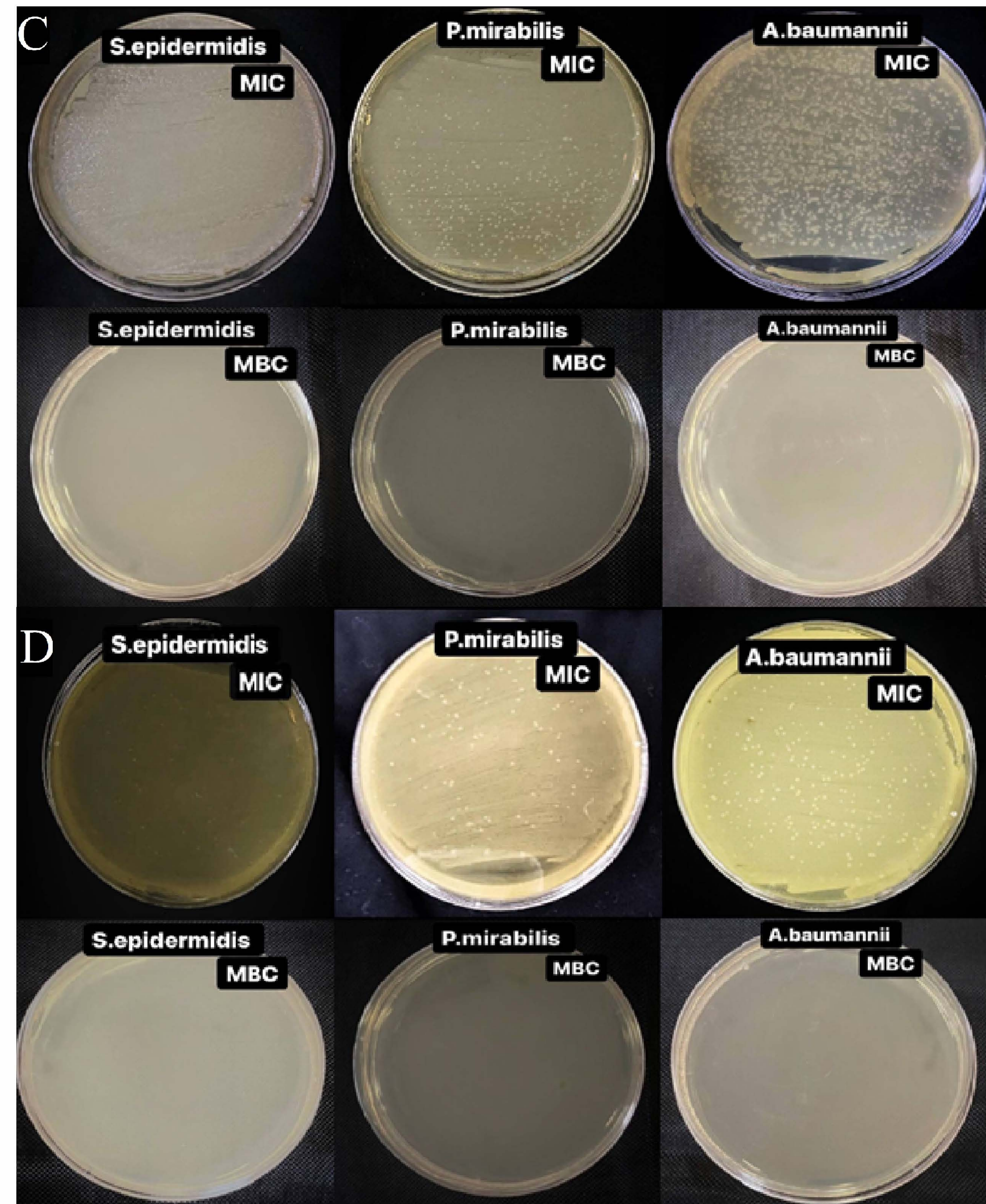

Supplementary Figure S1

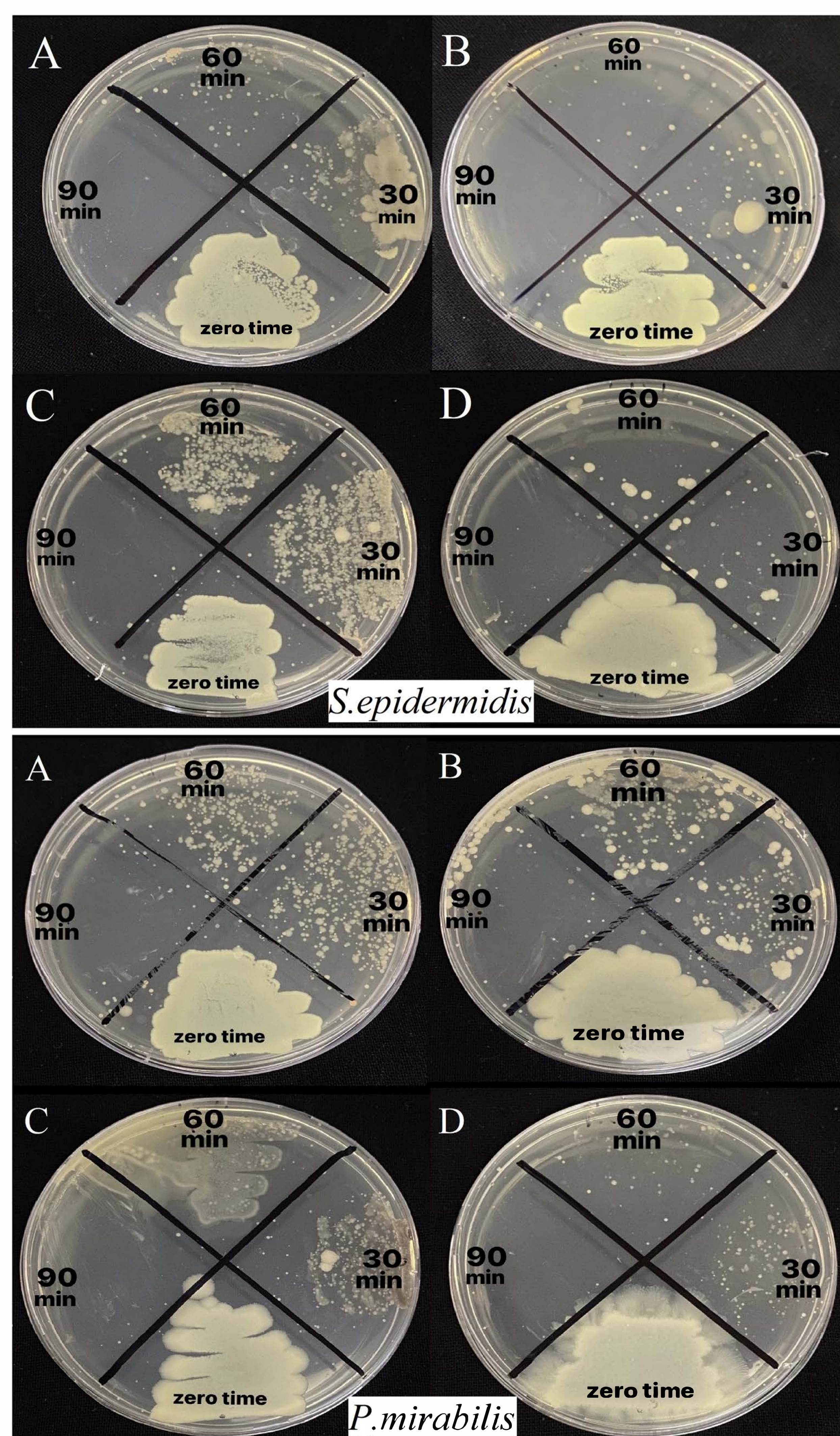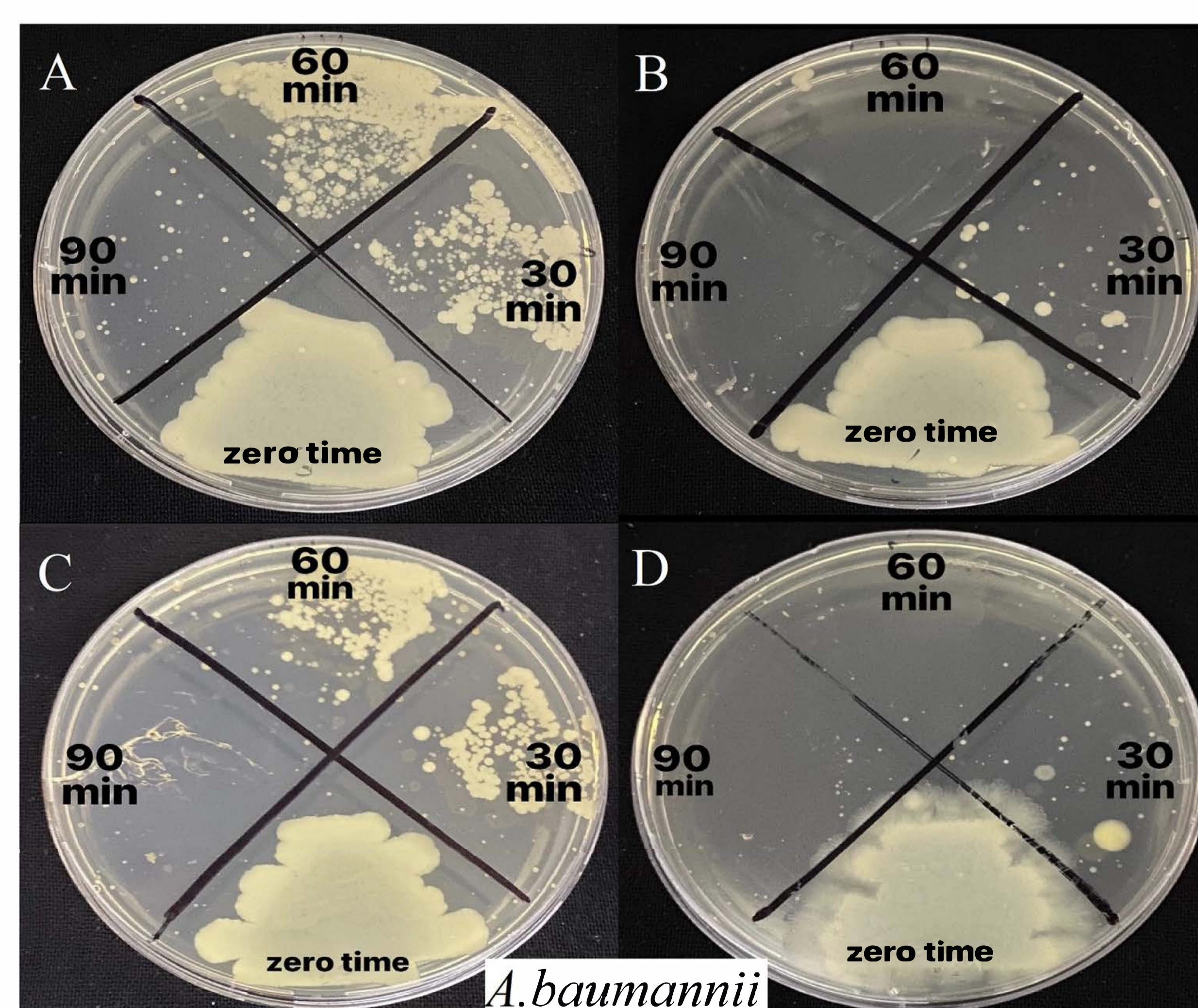

Supplementary Figure S2

Supplement: Supplementary file 1 [file jfb-13-00138-s001.zip › jfb-1881947-supplementary.pdf]
